# Age-Associated Epigenetic Upregulation of the FKBP5 Gene Selectively Impairs Stress Resiliency

**DOI:** 10.1371/journal.pone.0107241

**Published:** 2014-09-05

**Authors:** Jonathan J. Sabbagh, John C. O'Leary, Laura J. Blair, Torsten Klengel, Bryce A. Nordhues, Sarah N. Fontaine, Elisabeth B. Binder, Chad A. Dickey

**Affiliations:** 1 Department of Molecular Medicine, Byrd Alzheimer's Research Institute, University of South Florida, Tampa, Florida, United States of America; 2 Department of Psychiatry and Behavioral Sciences, Emory University School of Medicine, Atlanta, Georgia, Unites States of America; 3 Department of Translational Research, Max Planck Institute of Psychiatry, Munich, Germany; Rikagaku Kenkyūsho Brain Science Institute, Japan

## Abstract

Single nucleotide polymorphisms (SNPs) in the FK506 binding protein 5 (*FKBP5*) gene combine with traumatic events to increase risk for post-traumatic stress and major depressive disorders (PTSD and MDD). These SNPs increase FKBP51 protein expression through a mechanism involving demethylation of the gene and altered glucocorticoid signaling. Aged animals also display elevated FKBP51 levels, which contribute to impaired resiliency to depressive-like behaviors through impaired glucocorticoid signaling, a phenotype that is abrogated in *FKBP5^−/−^* mice. But the age of onset and progressive stability of these phenotypes remain unknown. Moreover, it is unclear how *FKBP5* deletion affects other glucocorticoid-dependent processes or if age-associated increases in FKBP51 expression are mediated through a similar epigenetic process caused by SNPs in the *FKBP5* gene. Here, we show that FKBP51-mediated impairment in stress resiliency and glucocorticoid signaling occurs by 10 months of age and this increased over their lifespan. Surprisingly, despite these progressive changes in glucocorticoid responsiveness, *FKBP5^−/−^* mice displayed normal longevity, glucose tolerance, blood composition and cytokine profiles across lifespan, phenotypes normally associated with glucocorticoid signaling. We also found that methylation of *Fkbp5* decreased with age in mice, a process that likely explains the age-associated increases in FKBP51 levels. Thus, epigenetic upregulation of FKBP51 with age can selectively impair psychological stress-resiliency, but does not affect other glucocorticoid-mediated physiological processes. This makes FKBP51 a unique and attractive therapeutic target to treat PTSD and MDD. In addition, aged wild-type mice may be a useful model for investigating the mechanisms of *FKBP5* SNPs associated with these disorders.

## Introduction

Late-life depression (LLD) is a debilitating disorder that can accelerate aging and mortality [1]–[3]. LLD is associated with reduced stress resiliency [4], a phenomenon that is influenced by the FK506 binding protein 51 (FKBP51) [5], [6]. Importantly, FKBP51 levels increase with age in humans and mice [7], [8]. This raises the possibility that senescence-related deficits in stress resiliency or coping ability may be due in part to increasing FKBP51 expression with age. Moreover, single nucleotide polymorphisms (SNPs) in the *FKBP5* gene that increase its expression through a mechanism involving demethylation increase susceptibility to anxiety disorders, post-traumatic stress disorder (PTSD) and major depression [9]–[12], further implicating a role for increased levels of this protein in psychiatric illnesses.

A role for FKBP51 in stress-related disorders was first suggested by its functional control of glucocorticoid receptor (GR) signaling [13]. FKBP51 has an inhibitory effect on GR, decreasing the affinity of cortisol or corticosterone (CORT) for GR [14] and impairing GR translocation [15]. This inhibition delays negative feedback of the hypothalamic-pituitary-adrenal (HPA) axis, prolongs the stress response and leads to elevated circulating CORT levels and glucocorticoid resistance. Previous work from our laboratory and others has shown that ablation of *FKBP5* in mice leads to an age-dependent anti-depressive phenotype, more robust coping behavior following stress, and reduced stress-induced circulating CORT levels without altering cognitive function [5], [6], [16]. *FKBP5^−/−^* mice are more responsive to the dexamethasone suppression test, suggesting HPA axis negative feedback is accelerated in the absence of FKBP51 [6]. Normalizing the HPA axis hyperactivity observed in depressed patients may be of critical importance for treatment outcome; individuals that display persistently elevated cortisol levels following treatment have higher rates of relapse [17]–[19]. Therefore, attenuating the effect of FKBP51 on HPA axis dysregulation is an attractive therapeutic strategy for depression, especially in aged individuals.

The current study sought to determine the effects of FKBP51 on behavioral and neuroendocrine changes across lifespan. We also examined whether FKBP51 affects stress-related phenotypes such as mortality, glucose tolerance, blood composition, cognitive flexibility, and inflammatory markers. Our data demonstrate that *FKBP5* deletion prevents the progressive age-associated increases in depression-like behavior and circulating CORT levels observed in wild-type mice. Furthermore, *FKBP5^−/−^* mice exhibited normal longevity, glucose tolerance, blood composition, and cytokine levels across lifespan, while even displaying enhanced cognitive flexibility in a reversal paradigm. We also show for the first time that wild-type mice display decreased DNA methylation of *Fkbp5* with age, modeling human disease. These findings support the viability of FKBP51 as a therapeutic target to treat stress-related disorders and suggest aged wild-type mice may have utility for studying SNP-like epigenetic changes in *FKBP5*.

## Results

### *Fkbp5* deletion reduced depression-like behavior and serum CORT levels across lifespan

**Figure 1:**
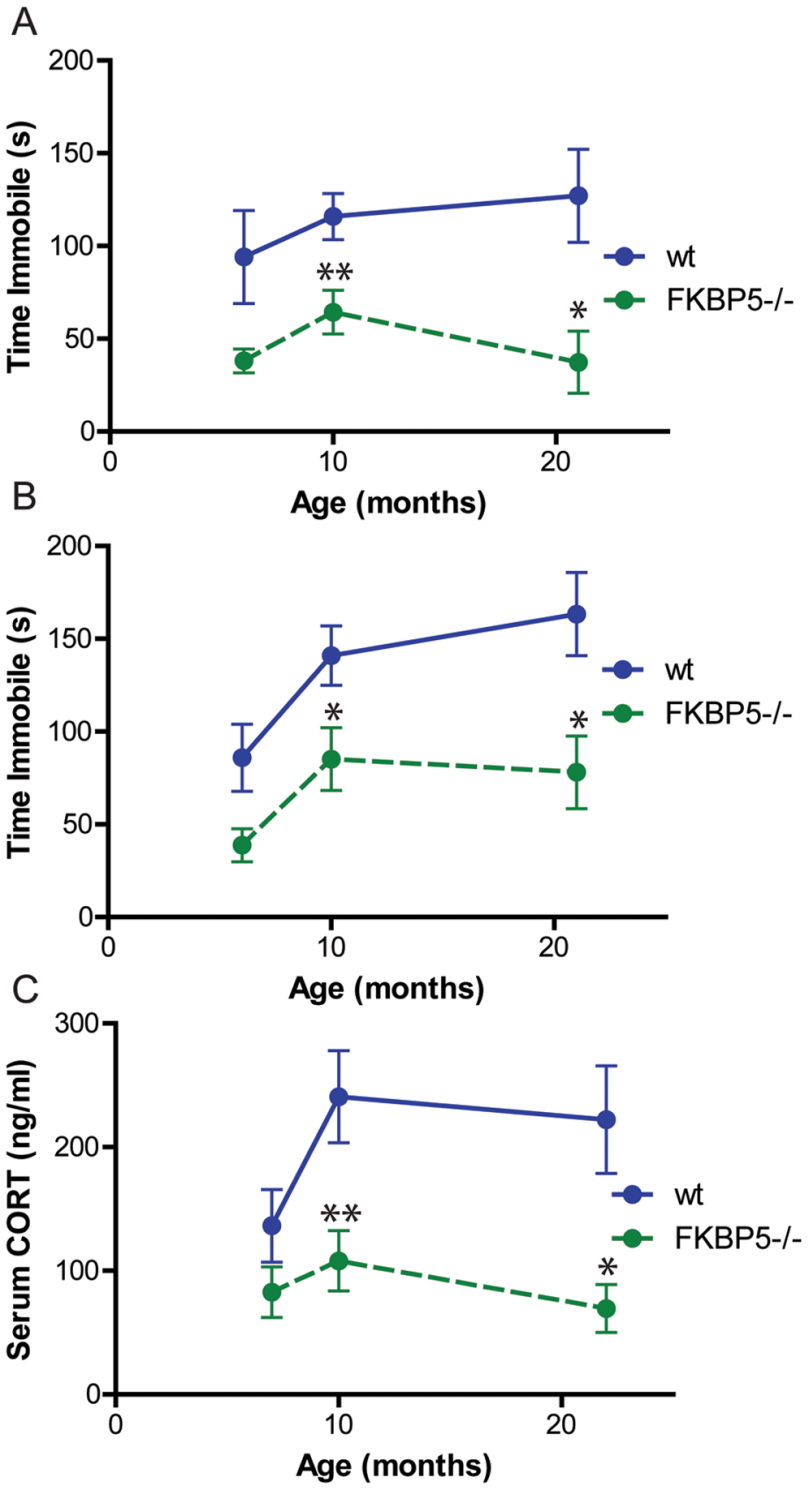
Ablation of *FKBP5* reduces depressive-like behavior and serum corticosterone (CORT) levels across lifespan. (A) *FKBP5^−/−^* mice display reduced immobility time in the forced swim test at 10 and 21 months. (B) *FKBP5^−/−^* mice have reduced immobility time in the tail suspension test at 10 and 21 months. (C) *FKBP5^−/−^* mice display reduced serum CORT levels following acute restraint stress at 10 and 21 months. **p<0.01, *p<0.05 as measured by two-way ANOVA. wild-type (wt). FST: n at 6 months: wt = 9, *FKBP5^−/−^* = 10; n at 10 months: wt = 30, *FKBP5^−/−^* = 33; n at 21 months: wt = 9, *FKBP5^−/−^* = 9. TST: n at 6 months: wt = 7, *FKBP5^−/−^* = 9; n at 10 months: wt = 18, *FKBP5^−/−^* = 19; n at 21 months: wt = 9, *FKBP5^−/−^* = 9. CORT ELISA: n at 6 months: wt = 8, *FKBP5^−/−^* = 10; n at 10 months: wt = 12, *FKBP5^−/−^* = 13; n at 21 months: wt = 5, *FKBP5^−/−^* = 6.

*FKBP5^−/−^* mice have previously demonstrated protection from depression-like behavior at 17–20 months of age [5], but not at 10–16 weeks [6]. Because FKBP51 levels increase with age, we hypothesized that a critical load of FKBP51 was necessary to produce this phenotype, but this was not known. To determine the age at which FKBP51 levels were sufficient to cause depression-like behavior and whether this phenotype was dependent on FKBP51 across lifespan, separate cohorts of wild-type and *FKBP5^−/−^* mice were examined at 6, 10, and 21 months of age for depression-like behavior in the forced swim test (FST) and tail suspension test (TST) and stress reactivity via post-restraint CORT sampling. At 6 months, no differences were found in the FST (Figure 1A), TST (Figure 1B), or in serum CORT levels (Figure 1C). However, at 10 months, increased immobility in the FST and TST, as well as elevated serum CORT levels following restraint stress emerged in wild-type mice, but not in *FKBP5^−/−^* mice (Figure 1A–C). These phenotypes remained in 21 month old wild-type mice, but again *FKBP5^−/−^* mice were protected. In fact, across whole lifespan, *FKBP5* ablation significantly reduced serum CORT levels (p<0.001) and immobility time in the FST (p<0.0001) and TST (p<0.001) as measured by two-way analysis of variance (ANOVA). Collectively, these data suggest that FKBP51 levels reach a critical load somewhere around 10 months of age, culminating in slowed negative feedback of the HPA axis, higher circulating CORT levels, and increased vulnerability to depressive-like phenotypes.

### *FKBP5* deletion does not impact other GR-dependent physiologies

**Figure 2:**
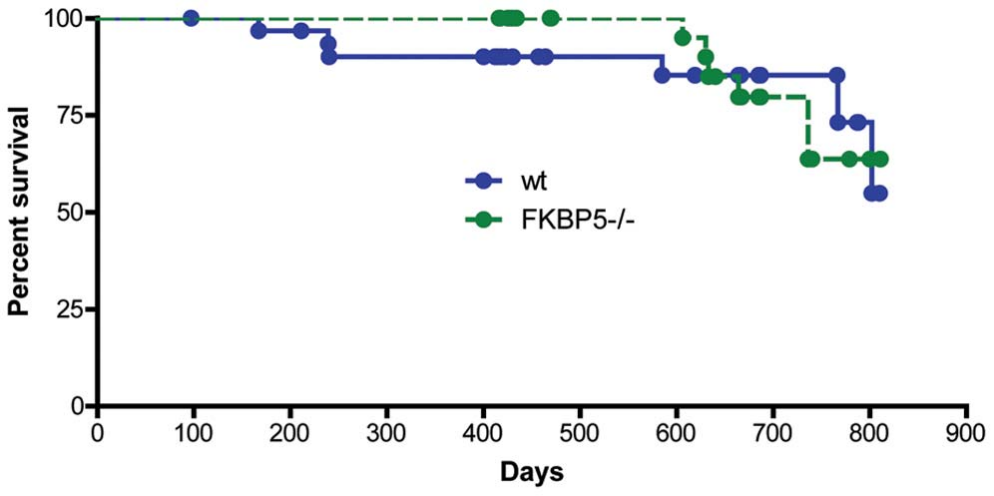
FKBP51 does not affect longevity. No significant differences were found in the percent survival of wild-type (wt) and *FKBP5^−/−^* mice, p>0.05. wt, n = 34 (18 male and 16 female); *FKBP5^−/−^*, n = 32 (18 male and 14 female).

Based on these results, we speculated that *FKBP5* deletion would impact other phenotypes related to HPA axis regulation. First, since the degree of HPA axis hyperactivity correlates with overall lifespan in rodents [20], such that shorter-lived strains are more reactive to stress and have elevated circulating glucocorticoids compared to longer-lived ones, we suspected that *FKBP5^−/−^* mice might have a longer lifespan than wild-type littermates. However, when we examined the survival curves of wild-type and *FKBP5^−/−^* mice, no differences were found in mouse survival up to 28 months of age, suggesting that FKBP51 neither increases or decreases longevity up to this point (Figure 2); but it is possible that differences between genotypes may emerge if animals are permitted to age longer.

**Figure 3:**
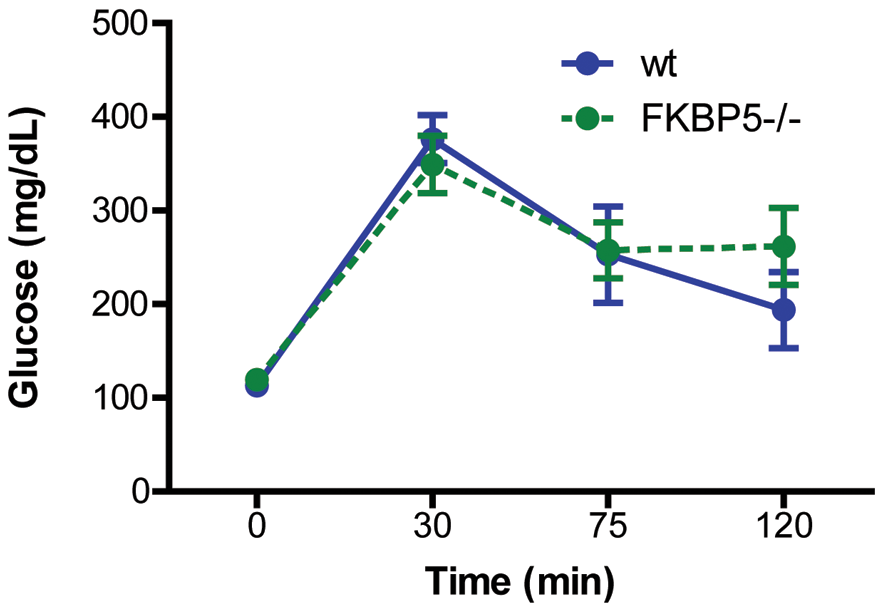
Deletion of *FKBP5* does not alter glucose metabolism. *FKBP5^−/−^* mice displayed normal glucose tolerance up to 120 minutes following glucose injection compared to wild-type (wt) mice, p>0.05. wt, n = 7; *FKBP5^−/−^*, n = 10.

Next, since GR signaling and FKBP51 have a prominent role in energy metabolism [21], we examined the effects of *FKBP5* deletion on glucose metabolism. A glucose tolerance test was performed in 10.5 month old wild-type and *FKBP5^−/−^* mice, wherein blood glucose levels were measured out to 120 minutes after glucose injection. No significant differences in glucose metabolism were observed between genotypes (p>0.05; Figure 3). Moreover, *FKBP5* deletion did not produce any deleterious effects on blood composition compared to wild-type mice. As shown in Table 1, there were no differences between genotypes in mass, the composition of red or white blood cells, or platelets.

**Table 1:** Blood composition and animal mass of wild-type versus FKBP5^−/−^ mice do not differ.

	Wild-type	*FKBP5^−/−^*	P value
Animal Mass (g)	43.04±3.35	38.82±2.32	0.3043
WBC (10^3^/µl)	4.16±0.69	3.30±0.82	0.4394
RBC (10^6^/µl)	7.05±0.89	4.76±0.90	0.0852
Lym (10^3^/µl)	3.12±0.53	2.43±0.62	0.4155
Mono (10^3^/µl)	0.32±0.04	0.24±0.04	0.2013
Gran (10^3^/µl)	0.72±0.13	0.63±0.17	0.6726
HGB (g/dl)	11.02±1.37	7.52±1.42	0.0921
PLT (10^3^/µl)	569.3±84.57	445.70±105.97	0.3779

WBC: white blood cells, RBC: red blood cells, Lym: lymphocytes, Mono: monocytes, Gran: granulocytes, HGB: hemoglobin, PLT: platelets. Wild-type, n = 11; FKBP5^−/−^, n = 12. Values are listed as the mean ± the standard error of the mean.

**Figure 4:**
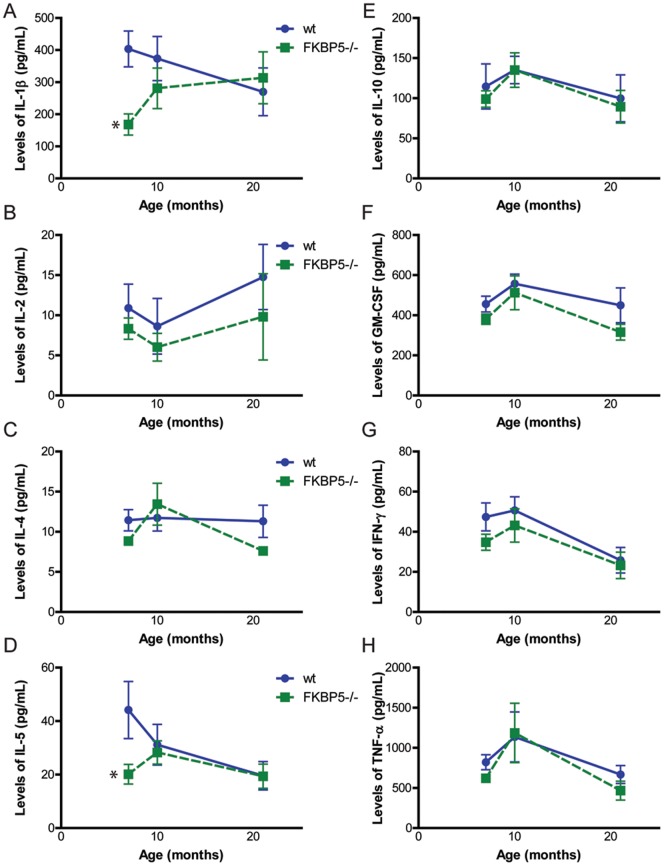
Ablation of *FKBP5* does not alter cytokine levels over time. Serum levels of interleukin-1β (A) and interleukin-5 (D) were decreased at 6 months (p<0.05 via t-test) but not across time (p>0.05 by two-way ANOVA). Levels of interleukin-2 (B), interleukin-4 (C), interleukin-10 (E), granulocyte-macrophage colony-stimulating factor (F), interferon gamma (G), or tumor necrosis factor alpha (H) did not differ between genotypes across lifespan, p>0.05. *p<0.05. wild-type (wt), n = 6 for each age; *FKBP5^−/−^*, n = 7 at 7 and 10 months, n = 6 at 21 months.

We then suspected that FKBP51 could impact immune function and inflammation given its role as an immunophilin and the known ability of GR to regulate transcription of inflammatory mediators [22]. Serum cytokines known to be affected by GR were examined from 6, 10, and 21 month old wild-type and *FKBP5^−/−^* mice (Figure 4). Levels of interleukin (IL)-1β (p<0.01; Figure 4A) and IL-5 (p<0.05; Figure 4D) were decreased at 6 months as measured by t-test but no other differences were observed. Across lifespan, no changes were found in any of the markers as measured by two-way ANOVA (p>0.05), suggesting the absence of FKBP51 does not adversely affect basal immune function. Taken together, these findings suggest that *FKBP5* deletion selectively protects aging mice from depressive-like behavior without impacting other important GR-mediated physiological effects.

### *FKBP5* deletion enhanced cognitive flexibility

**Figure 5:**
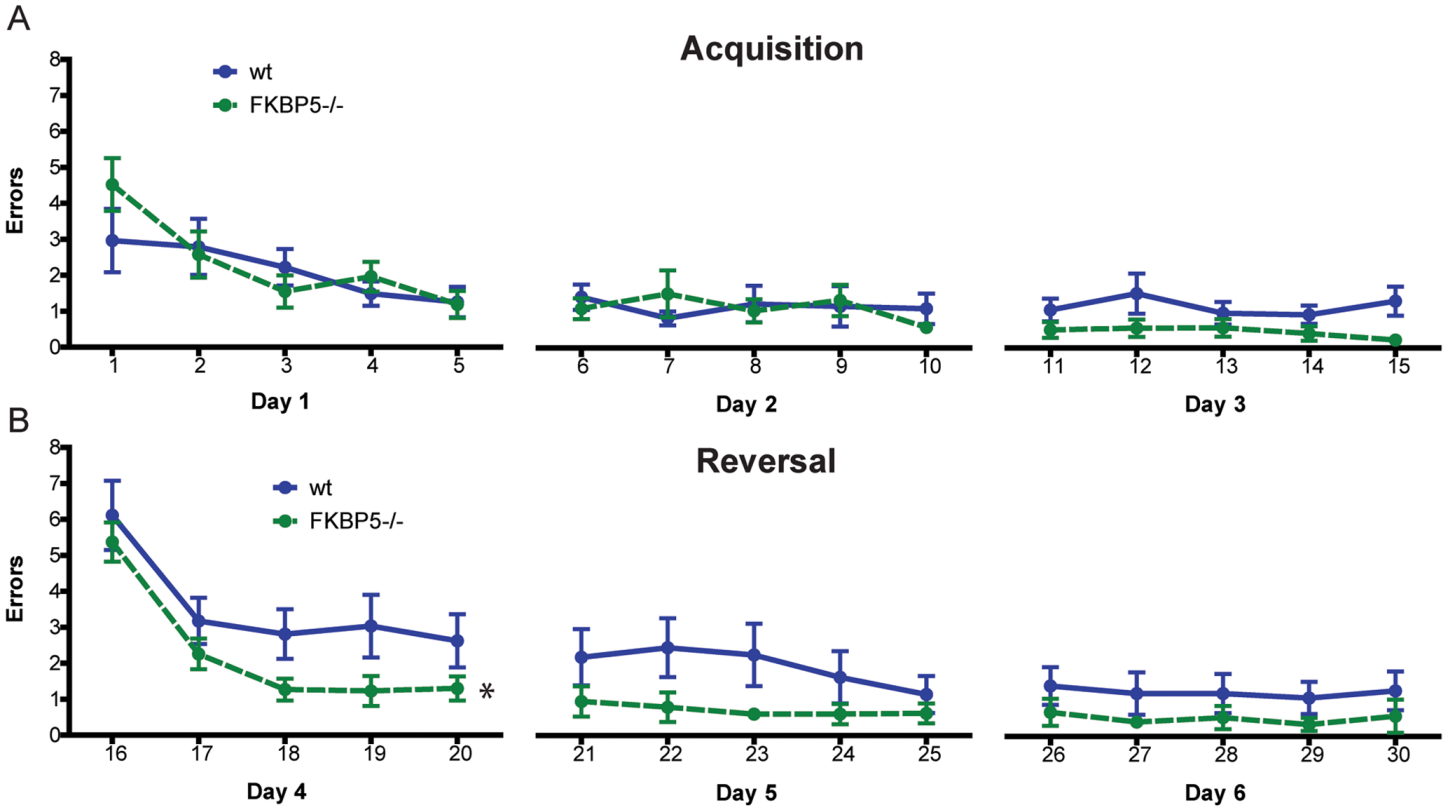
*FKBP5^−/−^* mice display enhanced cognitive flexibility in the radial arm water maze. (A) No differences were found in acquisition learning between genotypes (p>0.05). (B) There was a main effect of genotype (p<0.05) during reversal training, indicating *FKBP5^−/−^* mice made fewer errors across sessions. Data points represent a session of three trials. *p<0.05. wild-type (wt), n = 9; *FKBP5^−/−^*, n = 10.

*FKBP5^−/−^* mice do not display any cognitive deficits [5]; however, given the role of FKBP51 in resiliency, we suspected that these mice might be able to adapt better to a changing environment or paradigm. To test this, 6 month old mice were trained for three days to find the location of a hidden platform in the radial arm water maze (RAWM). They were then trained for three additional days to locate a hidden platform placed 180° from the original location. Although there were no differences in RAWM acquisition training to find the first location of the platform (p>0.05; Figure 5A), *FKBP5^−/−^* mice were able to find the new location of the platform significantly faster than wild-type littermates as measured by two-way ANOVA (p<0.05; Figure 5B). Thus, FKBP51 can interfere with stress-based learning and cognitive flexibility. These findings indicate that increasing levels of FKBP51 exacerbate stress-responsiveness, particularly to psychological stimuli, to the point of pathogenesis. While a degree of FKBP51 expression may be necessary for threat assessment [23], overabundance of FKBP51 can be problematic. Therefore, we next sought to examine how *FKBP5* expression was increasing with age in an effort to identify ways to reduce the expression of this gene.

### The *Fkbp5* gene is demethylated with age

**Figure 6:**
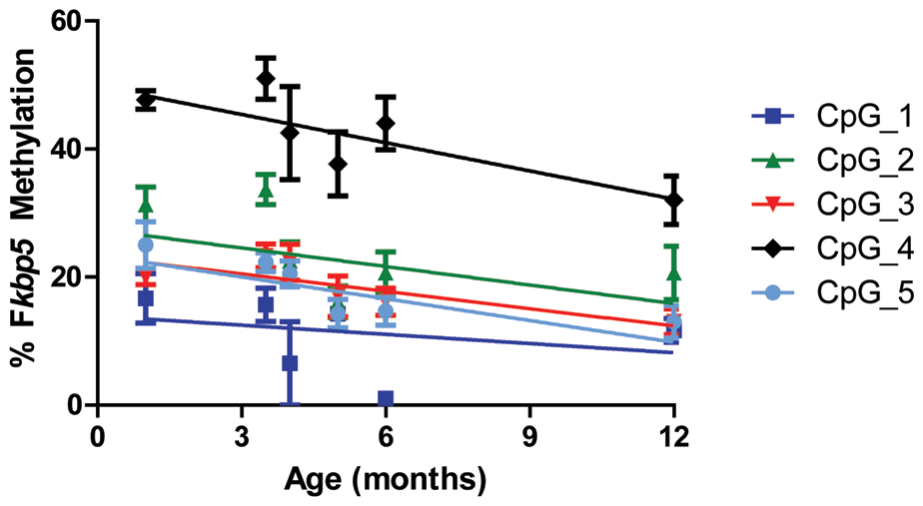
*Fkbp5* DNA methylation decreases with age in mice. Isolated DNA samples from wild-type mice aged 1 (n = 3), 3.5 (n = 3), 4 (n = 6), 5 (n = 7), 6 (n = 6), and 12 (n = 3) months were subjected to bisulfite pyrosequencing. Multiple CpG sites in intron 5 were analyzed for *Fkbp5* methylation. Significant demethylation was found at CPG_3 (r = −0.3890, p<0.05), CPG_4 (r = −0.4004, p<0.05) and CpG_5 (r = −0.5044, p<0.01) as measured by linear regression analyses.

Several studies have shown that SNPs linked to the *FKBP5* gene or stress each increase FKBP51 expression through a process involving demethylation of the *FKBP5* gene. We suspected that this same mechanism was responsible for the increased expression of FKBP51 in aging mice. We performed bisulfite pyrosequencing on isolated DNA from wild-type mouse frontal cortex to examine age-dependent *Fkbp5* methylation in intron 5, which contains a functional glucocorticoid response element (GRE) [24], [25]. As expected, linear regression analyses revealed that *Fkbp5* methylation decreased with age at multiple CpG sites (CPG_3: r = −0.3890, p<0.05; CPG_4: r = −0.4004, p<0.05; CpG_5: r = −0.5044, p<0.01; Figure 6). Demethylation in intron 5 has been shown to increase *Fkbp5* mRNA expression [25], suggesting this epigenetic phenomenon is also likely responsible for the age-related increase in FKBP51 levels previously observed in mice [8].

## Discussion

The *FKBP5* gene is associated with depression [10], PTSD [9], anxiety [11], [26] and Alzheimer's disease (AD) [7], [8], but little is known about its role in aging. Here we demonstrate that the abundance of FKBP51 by 10 months of age is sufficient to increase depression-like behavior and stress-induced serum CORT levels without adversely affecting other glucocorticoid-dependent processes. Our data also show for the first time a learning enhancement following *FKBP5* deletion in a task dependent upon cognitive flexibility in a stressful environment. Finally, we have shown progressive *Fkbp5* demethylation occurs with age in wild-type mice, elucidating a mechanism by which FKBP51 levels increase throughout life. These findings suggest that aging acts as an epigenetic milieu similar to how SNPs interact with early life events to promote vulnerability to depression and other disorders.

Prolonged exposure to glucocorticoids is considered a potential driving factor for depression [27], suggesting elevations in FKBP51 due to age or SNPs could facilitate onset of the disorder. Our data demonstrated that wild-type mice have an age-dependent increase in depression-like behavior and circulating CORT levels that was absent in *FKBP5^−/−^* mice, indicating these age-related changes were mediated by increasing FKBP51 expression with age. Glucocorticoid signaling is also vital for a number of physiological processes, raising the possibility that deleting *FKBP5* could affect some of these pathways. For example, longevity can be influenced by stress and depression [2], [3], [28], [29], leading us to hypothesize that reducing FKBP51 levels could be beneficial for survival. Aberrant glucose metabolism is observed in patients with major depression and Cushing's disease and following chronic CORT exposure in rodents [30]–[32]. Furthermore, overexpression of FKBP51 in the hypothalamus impaired glucose tolerance, while food deprivation increased *Fkbp5* mRNA expression in mice [21], suggesting FKBP51 may play a role in energy metabolism. Finally, because FKBP51 is an immunophilin and it has been implicated in the dysregulation of nuclear factor kappa-light-chain enhancer of activated B cells (NF-κB) signaling [33], its deletion could alter levels of GR-regulated inflammatory markers. However, our data reveal that *FKBP5^−/−^* mice display normal longevity, glucose tolerance, blood composition, and cytokine profile. These findings are important for determining the potential adverse effects of therapeutically targeting FKBP51.

Thus far FKBP51 has not been implicated in the regulation of synaptic plasticity or learning. However, its expression has been linked to EphB2/NR1 signaling [26], while others have shown that individuals with the rs1360780 *FKBP5* SNP have altered hippocampal function and morphology [23]. We have previously shown that deletion of *FKBP5* does not affect basic hippocampal function in mice [5], but we predicted that perhaps a behavior influenced by stress could be modulated by FKBP51. Using a reversal paradigm in the RAWM, we were able to discriminate differences in behavior between wild-type and F*KBP5^−/−^* mice. In fact, *FKBP5^−/−^* mice made fewer errors than wild-type mice, indicative of enhanced cognitive flexibility. Chronic stress and aging have been demonstrated to independently impair cognitive flexibility in rodents and humans [34]–[37]. Together with our data, these findings suggest progressively increasing FKBP51 expression with age may attenuate the ability to rapidly shift strategy and retain cognitive flexibility under stressful conditions.

While demethylation of *FKBP5* has been identified as a potential mechanism for FKBP51 upregulation in humans [7], [12], it was not known if this was contributing to the progressive increases in FKBP51 that we observed in aged mice [8]. We demonstrated that *Fkbp5* methylation decreases with age in wild-type mice, underscoring the validity of utilizing aged mouse models to study *Fkbp5* gene x environment interactions. Interestingly, chronic CORT treatment decreases *Fkbp5* methylation in mice similar to aging [25], linking the effects of stress and aging through FKBP51. Previous work has shown that functional SNPs in *FKBP5* lead to demethylation of the gene in an interaction with early environmental factors [12]. Our data suggest that aging acts similarly to the SNPs, leading to epigenetic changes in *FKBP5* regulation across lifespan. Because GR stimulation leads to *FKBP5* demethylation [12], [38], it is possible that stress throughout life may lead to progressively decreased methylation and difficulty regulating the HPA axis and the stress response with age.

Elderly depressed patients suffer from increased treatment resistance and frequency of depressive episodes [39], [40]. Depression is frequently comorbid with AD and may be a risk factor for development of AD [41], [42]. Moreover, expression of *FKBP5* increases with age and is even further elevated in AD, providing a putative link between LLD and AD [7], [8]. Therefore, inhibition of FKBP51 is a desirable strategy for stress-related disorders such as depression and age-related diseases. Although the development of FKBP51 ligands has been difficult due to the similarity between FKBP51 and its homologues, it is considered a tractable, viable drug target [43]. Our data support the need for continued research into FKBP51 biology and the advancement of its therapeutic development. Furthermore, we provide data supporting the idea that aging activates an epigenetic mechanism that increases FKBP51 expression, leading to impaired HPA axis function and LLD-like phenotypes. Thus, aged wild-type mice may model human conditions caused by SNPs in the *FKBP5* gene.

## Materials and Methods

### Animals and experimental design

*FKBP5^−/−^* and wild-type littermate mice were generated and genotyped as described previously [5]. All animals were naïve to each procedure performed. For *Fkbp5* methylation studies, a separate cohort of wild-type mice aged to 1, 3.5, 4, 5, 6, or 12 months of age was used. Longevity was compared between wild-type and *FKBP5^−/−^* mice in a subset of animals used for behavioral analyses. Glucose tolerance was measured in 10.5 month old mice that had previously been tested at 6 months of age in the TST, FST, and the RAWM. The complete blood composition analysis was performed in a separate cohort of 14 month old mice that were previously tested in the TST and FST at 10 months of age. A final cohort was tested in the TST and FST at 21 months of age. CORT and cytokine levels were examined from the serum of animals used for behavioral analyses. All animal studies were approved by the University of South Florida Institutional Animal Care and Use Committee and carried out in accordance with the National Institutes of Health (NIH) guidelines for the care and use of laboratory animals. Mice were group housed under a 12 hour light-dark cycle (lights on at 06:00) and permitted *ad libitum* access to food and water.

### DNA methylation analyses

DNA was isolated from 15 mg of flash frozen frontal cortex brain tissue from wild-type mice aged 1, 3.5, 4, 5, 6 and 12 months old using a QuickGene DNA tissue kit (Autogen; Holliston, MA). Briefly, tissue was incubated overnight on a shaking incubator at 70°C in tissue lysis buffer supplemented with Proteinase K (Autogen). DNA was isolated following complete tissue lysis as directed by the manufacture, with RNase treatment to remove residual RNA. Isolated DNA was purified through provided columns in a QuickGene Mini80 (Autogen). Following isolation, DNA concentration and purity were determined using a Qubit 1.0 Fluorometer (Invitrogen; Grand Island, NY) and Nanodrop (Thermo Scientific; Wilmington, DE), confirmed by visualization on a 1% agarose DNA gel. Pure DNA was then subjected to bisulfite pyrosequencing as previously described [7], [12]. Mouse *Fkbp5* DNA was evaluated at 5 CpG sites across intron 5 [25]. Specifically, DNA methylation was interrogated in the intron 5 region of mouse *Fkbp5* harboring functional GREs and previously shown to be demethylated in response to GR activation [25]. Sequenom EpiTYPER DNA methylation analysis was performed according to the instructions of the manufacturer. Primer sequences for amplification of a 412 bp *Fkbp5* fragment from bisulfite converted DNA were: F_mouse-I5 5′-aggaagagagAATATTTTGTTTTGAATGTGGTTGG-3′ and R_mouse-I5 5′- cagtaatacgactcactatagggagaaggctCTCTCCTAAAAACCCTTACCCAATA-3′. Uncertainty threshold was set to 0.1.

### Survival analysis

To measure longevity, an independent cohort of wild-type and *FKBP5^−/−^* mice was monitored until death or euthanasia was deemed necessary by veterinarian assessment. The survival curve was plotted using the Kaplan-Meier method and statistically analyzed by GraphPad Prism software. Animals were aged to 28 months of age, at which point all surviving animals were humanely euthanized.

### Glucose tolerance test

Mice were fasted overnight by removing food at 18:00. The following day, animals were injected intraperitoneally with glucose (2 g/kg) and blood was extracted from the tail vein prior to and 30, 75, and 120 minutes after injection. Blood glucose levels were measured with a True Track blood glucose meter (NIPRO Diagnostics, Fort Lauderdale, FL).

### Radial arm water maze

The RAWM was adapted from previous studies [44]. Briefly, a circular black tank with a six arm metal insert was filled with water. A platform was submerged 1 cm below the surface of the water at the end of a designated goal arm. Animals were permitted 60 seconds to locate the platform during which time an observer blind to genotype manually scored the number of errors. An error was defined as an entry into an incorrect arm. Mice were trained for 15 trials per day, which were divided into 5 sessions of 3 trials each. Following 3 days of acquisition training with a consistent platform location, the platform was moved 180° to test reversal learning, a measure of cognitive flexibility.

### Tail suspension test

The TST was conducted as previously described [5]. Mice were suspended from the tail for one 6 minute session which was video recorded (ANY-maze Software, Stoelting Co., Wood Dale, IL). The amount of time spent immobile was recorded by a trained observer blind to genotype.

### Forced swim test

The FST was performed as previously reported [5]. Mice were placed in a clear glass cylinder 23 cm high and 15 cm wide, filled with room temperature water to a depth of 13 cm. Each session lasted 6 minutes and was video recorded. The amount of time spent immobile was measured by a trained observer blind to genotype.

### Enzyme-linked immunosorbent assays

Blood was collected from mice through the submandibular vein one hour after the start of the light cycle. For measurement of CORT, blood was collected 30 minutes following a 10 minute tube restraint. Serum was separated from blood using BD Microtainer serum separator tubes (BD Biosciences, Sparks, MD) and centrifuged at 1,300× g. CORT levels were measured using a CORT enzyme-linked immunosorbent assay (ELISA) kit (Enzo Life Sciences, Farmingdale, NY) and cytokines were analyzed with a Bio-Plex Pro Mouse Cytokine 8-plex Assay (Bio-Rad, Hercules, CA) according to manufacturer's instructions.

### Statistical analyses

Statistical significance for each analysis was determined with linear regression, Student's *t* tests, or two-way ANOVA with Bonferroni post-tests to compare groups where appropriate. All figures and statistics were generated using GraphPad Prism software; each graph represents the mean ± the standard error of the mean (SEM).
